# American Indian Parents’ Assessment of and Concern About Their Kindergarten Child’s Weight Status, South Dakota, 2005-2006

**Published:** 2012-02-16

**Authors:** Chrisa Arcan, Peter J. Hannan, John H. Himes, Jayne A. Fulkerson, Bonnie Holy Rock, Mary Smyth, Mary Story

**Affiliations:** University of Minnesota School of Public Health, Division of Epidemiology and Community Health; Division of Epidemiology and Community Health, School of Public Health, University of Minnesota, Minneapolis, Minnesota; Division of Epidemiology and Community Health, School of Public Health, University of Minnesota, Minneapolis, Minnesota; School of Nursing, University of Minnesota, Minneapolis, Minnesota; Division of Epidemiology and Community Health, School of Public Health, University of Minnesota, Minneapolis, Minnesota; Division of Epidemiology and Community Health, School of Public Health, University of Minnesota, Minneapolis, Minnesota; Division of Epidemiology and Community Health, School of Public Health, University of Minnesota, Minneapolis, Minnesota

## Abstract

**Introduction:**

Obesity is highly prevalent among American Indians, and effective prevention efforts require caregiver involvement. We examined American Indian (AI) parents' assessment of and level of concern about their kindergarten child's weight status.

**Methods:**

We collected baseline data (fall of 2005 and fall of 2006) on children and their parents or caregivers for a school-based obesity prevention trial (Bright Start) on an AI reservation in South Dakota. The current study uses 413 parent-child pairs. Age- and sex-adjusted body mass index percentiles were categorized as very underweight (<5th percentile), slightly underweight (5th to <15th percentile), normal weight (15th to <85th percentile), overweight (85th to <95th percentile), and obese (≥95th percentile). Parents or caregivers reported their assessment of and concerns about their child's weight status as well as sociodemographic characteristics. We used mixed-model multivariable analysis to examine associations between sociodemographic characteristics and the probability of parents underclassifying or overclassifying their child's weight status; analyses were adjusted for school as a random effect.

**Results:**

Children were evenly divided by sex and had a mean age of 5.8 years. Twenty-nine percent of children and 86% of parents were overweight or obese. Approximately 33% (n = 138) of parents underclassified and 7% (n = 29) of parents overclassified their child's weight status. Higher parental weight status and higher concern about their child's weight status increased the probability of underclassification (*P* for trend = .02 for both).

**Conclusion:**

In this sample of at-risk children, one-third of parents underclassified their child's weight status. Childhood obesity prevention programs need to increase awareness and recognition of childhood obesity and address parental weight issues.

## Introduction

Childhood overweight and obesity are prevalent throughout the United States, and racial/ethnic minorities are disproportionately affected (1). Among American Indian (AI) children, underweight and nutritional deficiencies that were prevalent 50 years ago have now been replaced by an epidemic of obesity (2,3). Increasing secular trends in overweight status among AI children were observed from 1955 through 1997; the prevalence of overweight was 41% in 1997 (4). More recent data indicate that even at age 5 years, 40% of AI children living on reservations were overweight or obese (5). In 2007, the age-adjusted percentage of AI adults who were obese was 33%, which was 1.3 times higher than that reported for non-Hispanic whites (6). An increase in the prevalence of type 2 diabetes has paralleled this trend (7).

Interventions to prevent or reverse the increasing obesity trend in children require family involvement, especially during the preschool years, because parents and caregivers shape childrens' environments and behavior, including opportunities for healthy eating and physical activity (8,9). In addition, school-aged children who are overweight and have obese parents have a more than 70% chance of being obese in young adulthood (10). Although studies have found mixed results regarding parents' body mass index (BMI) and assessment of their child's weight status (11,12), parents' ability to identify their own weight status may also help in correctly classifying their childrens' weight status (12). Parents must recognize their childrens' risk for obesity (ie, genetic, behavioral, and environmental) to be more actively engaged in the process of helping their children adopt healthy lifestyles.

Parents tend to misclassify their childrens' weight (13). Studies with children of all ages (≤18 y) and diverse racial/ethnic backgrounds found that only one-third or fewer parents correctly identified the weight status of their overweight or obese children (11,12,14-17). Among AI children aged 4.5 to 8.5 years residing on a reservation in Wisconsin, only 15% of caregivers of overweight children correctly classified their child's weight status (18).

Given the high prevalence of obesity and the related comorbidities among AI populations (2,3,7), it is necessary to understand parents' attitudes toward their childrens' weight status to more effectively engage parents in preventive efforts. Our objective was to examine parents' assessment of and concerns about their child's weight status and factors that are associated with these perceptions among a group of AI children and their parents and caregivers living on a reservation in South Dakota.

## Methods

The Bright Start study was a group-randomized controlled trial of an intervention to reduce excess weight gain through dietary and physical activity environmental changes among AI children residing on the Pine Ridge Reservation in South Dakota. We used a cross-sectional analysis of baseline data, collected in the fall of 2005 and fall of 2006, before children were randomly assigned to intervention and control groups. We conducted the trial in 14 schools; 7 were randomized to the intervention and 7 to control conditions. Of the 472 kindergarten children on the records of the 14 schools, we obtained consent for 99% of the children from parents or caregivers (hereafter referred to as parents), and 97% of those consenting agreed to participate in the study. A total of 454 children (96% of those eligible) had baseline measurements. Of these study children, 417 had a parent who completed a study survey that assessed sociodemographic characteristics and their own dietary and physical activity behaviors as well as those of their child. Both children and their parents had their heights and weights measured by trained research staff before survey completion. All study procedures were approved by the University of Minnesota's institutional review board (IRB) human subjects committee, the Oglala Sioux Tribe, and the Aberdeen Area IRB. This manuscript was also reviewed by the relevant tribal IRB committees.

### Measures

**Child and parent anthropometry**

We measured childrens' and parents' weight in kilograms to the nearest 100 g using a Tanita model 300 scale (Tanita Corp, Arlington Heights, Illinois); participants wore light indoor clothing. Height was measured to the nearest to 0.1 cm using a portable stadiometer (Perspective Enterprises, Portage, Michigan). Data were collected by trained study staff, according to protocols by Lohman and colleagues (19).

We calculated BMI as kilograms of weight divided by height in meters squared. For children, we created weight status categories using BMI percentiles adjusted for age and sex, derived from the 2000 Centers for Disease Control and Prevention Growth Charts (20). Very underweight was defined as BMI less than the 5th percentile; slightly underweight as BMI from the 5th to less than the 15th percentile; normal weight as BMI from the 15th to less than the 85th percentile; overweight as BMI from the 85th to less than the 95th percentile; and obese as BMI of 95th percentile or more. For only the description of parent concern and measured child weight, we created categories for obese (BMI 95th to <97th percentile) and extremely obese (BMI ≥97th percentile). For parents, normal weight was defined as BMI of less than 25.0, overweight as BMI of 25.0 to 29.9, and obese as BMI of 30.0 or more.

**Parent survey**

Parents' perception of their child's weight status was assessed with the question, "How would you describe your kindergarten child's weight?" (14). Response categories were very underweight, slightly underweight, about the right weight, slightly overweight, and very overweight. Parents were unaware of their child's measured height and weight. Parents reported their child's sex, and age at baseline was calculated using parent report of date of birth. Relationship to the child was assessed with the question, "Which of the following best describes your relationship to your kindergarten child?" Eleven response categories were consolidated to mother/stepmother, father/stepfather, grandmother, and other (grandfather, aunt, uncle, guardian/foster parent, adoptive parent, and other.) Three categories of parents' concern about their child's current weight were created: not concerned, a little concerned, and concerned or very concerned. Income was used as a measure of socioeconomic status, which is related to obesity prevalence (21,22). Parents reported on their family's income in the past year, and 3 categories were created that corresponded to the widespread poverty on the reservation: less than $15,000, $15,000 to $34,999, and $35,000 or more.

### Statistical analysis

We calculated frequencies of parents' misclassification of their child's weight status — the probability of either underclassifying or overclassifying their child's weight status. The outcome variable was derived by subtracting the child BMI percentile categories (very underweight, slightly underweight, normal weight, overweight, and obese from actual measured BMI) from the parent perception categories (very underweight, slightly underweight, about the right weight, slightly overweight, and very overweight.) Negative values represented parent underclassification, and positive values overclassification, of their child's weight status. We used mixed-model analysis of variance to examine cross-sectional associations between parents' underclassification or overclassification of their child's weight status with parent characteristics and demographic factors. All independent variables (child's sex, age, parent's/caregiver's relationship to child, parent's level of concern, parent's BMI, and family income) were simultaneously included in the model. The model was adjusted for school as a random effect, accounting for the additional component of variance associated with a cluster sampling design where observations from students from the same schools may be correlated (23). All analyses were completed using SAS 9.2 (SAS Institute, Inc, Cary, North Carolina).

## Results

There were 413 parent-child pairs; 51% of children were male, and 89% of parents were female. The mean (standard deviation [SD]) age of the children at baseline was 5.8 (0.5) (range, 4.7-7.9), and the mean age of the parents was 35.6 (10.8) (range, 19.2-74.0). Twenty-nine percent of the children were classified as overweight or obese. Most (86%) of the parents were overweight or obese; their mean (SD) BMI was 32.5 (7.3). The distribution of parents/caregivers were as follows: mothers, 68%; grandmothers, 15%; fathers/stepfathers, 9%; and other (8%).

Most parents (81%) classified their child as about the right weight, 9% classified their child as very underweight or slightly underweight, and 10% classified their child as very overweight or slightly overweight. Approximately 60% (n = 246) of parents correctly classified their child's weight status, 33% (n = 138) underclassified, and 7% (n = 29) overclassified (Table 1). Forty-six percent (n = 29) of parents of obese children and 90% (n = 55) of parents of overweight children classified their child as being the right weight, and approximately 9% (n = 22) of parents of normal-weight children classified their child as underweight. In contrast, 1.5% (n = 4) of parents classified their normal-weight child as slightly overweight.

**Figure 1.**
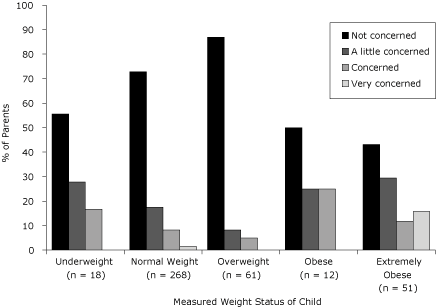
Parents' level of concern about their child's current weight status and child's measured weight among American Indians, South Dakota, 2005-2006. Weight status was based on body mass index percentiles from the 2000 Centers for Disease Control and Prevention Growth Charts (20). Very underweight: <5th percentile; underweight: 5th to <15th percentile; normal weight: 15th to <85th percentile; overweight: 85th to <95th percentile; obese: 95th to <97th percentile; extremely obese: ≥97th percentile. The categories "very underweight" and "underweight" were combined. Parents were unaware of child's measured height and weight.

**Figure 2.**
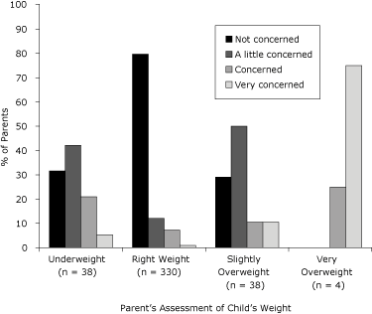
Parents' level of concern about their child's current weight status and assessment of child's weight among American Indians, South Dakota, 2005-2006. The categories "slightly underweight" and "very underweight" were combined.

More female children were correctly classified but more male children were underclassified by their parents (Table 2). Most parents who reported not being concerned about their child's current weight were parents of overweight followed by normal-weight children (Figure 1). Approximately 16% (n = 8) of parents of extremely obese children (BMI ≥97th percentile) reported being very concerned, while 50% (n = 6) and 43% (n = 22) of parents of obese and extremely obese children, respectively, reported not being concerned. When parents assessed their child's weight status, 75% (n = 3) of those reporting that their child was very overweight were very concerned, while 10.5% (n = 4) of those reporting that their child was slightly overweight were very concerned and 79% (n = 30) were a little or not concerned (Figure 2).

**Table:** 

Level of Concern	Measured Weight Status of Child				

	Underweight (n = 18)	Normal Weight (n = 268)	Overweight (n = 61)	Obese (n = 12)	Extremely Obese (n = 51)
**Not concerned, %**	55.6	72.8	86.9	50.0	43.1
**A little concerned, %**	27.8	17.5	8.2	25.0	29.4
**Concerned, %**	16.7	8.2	4.9	25.0	11.8
**Very concerned, %**	0	1.5	0	0	15.7

**Table:** 

Level of Concern	Parents’ Assessment of Child’s Weight			

	Underweight (n = 38)	Right Weight (n = 330)	Slightly Overweight (n = 38)	Very Overweight (n = 4)
**Not concerned, %**	31.6	79.7	29.0	0
**A little concerned, %**	42.1	12.1	50.0	0
**Concerned, %**	21.0	7.3	10.5	25.0
**Very concerned, %**	5.3	0.9	10.5	75.0

There was a 22% probability of normal-weight parents underclassifying their child's weight status; overweight parents had 32% probability, and obese parents had 42% probability of so doing (*P* for trend = .02) (Table 3). Being concerned about the child's weight status increased the probability of underclassification from 31% to 50% (*P* for trend = .02). There was a trend between family income and probability of a child being underclassified; children in the lowest family income category had 33% probability of being underclassified, and this probability increased to 49% at the highest income category (*P* for trend = .04). Regarding overclassifying their child's weight status, parents who were not concerned had a 7% probability, and those who were concerned or very concerned had a 13% probability of overclassifying their child's weight.

## Discussion

Our findings indicate that a large proportion of parents of overweight or obese children underclassified their child's weight status. However, parents' underclassification of their child's weight status is not unique to AI children; it has been observed in studies among other racial/ethnic groups (14,15,18,24-28). Studies with predominantly Hispanic and African American children aged 2 to 4 years participating in the Special Supplemental Nutrition Program for Women, Infants, and Children found that 79% to 93% of parents of obese children did not perceive their child as obese (14,25). Among obese or very obese African American children aged 5 to 10 years, only 44% of parents perceived their child's weight to be a problem (26).

In our study, only 6.3% of parents correctly identified their child as obese compared to 15.1% of parents in another study among American Indian children aged 4.5 to 8.5 years living on a reservation in Wisconsin (18). Our findings, coupled with an appreciable number of parents of normal-weight children who underclassified their child's weight status, are worrisome, considering the high rates of overweight and type 2 diabetes among American Indian children and adults (7). If parents of normal-weight children perceive their children as underweight, they may inadvertently encourage them to eat more, putting them at risk for becoming overweight.

In our study, only 4 parents perceived their child as very overweight, and 3 of those reported being very concerned. However when the child's measured weight status was in the extremely obese category (BMI ≥ 97th percentile; n = 51), only about 16% of parents reported being very concerned about their child's current weight status, despite being unaware of their child's measured weight status. Also, only 21% of parents who considered their child slightly overweight were concerned or very concerned. In addition, our findings show that as parents reported being more concerned, the probability of underclassifying their child's weight status increased. Collectively, these findings suggest that the parents in our study recognize their child's weight issue when their child is at the highest weight percentile, and they are more concerned when they perceive their child's weight status to be lower than the actual weight status. Our findings are consistent with other studies among preschool-age children (14,17,18,27).

Cultural, social, or environmental factors may influence parental perceptions of childrens' weight. Because of nutritional deficiencies that were prevalent among AI populations in previous generations, beliefs and worries about undernutrition may still be relevant and may result in a desire for children to weigh more to protect against malnutrition or illness (2). Cultural perspectives in relation to weight have been shown in other studies. Low-income Latino mothers in New York City expressed that more weight for their children is "safer" and is necessary for their "childrens' protection." They also indicated that "eating right" meant gratifying children with food and is an indication of "good parenting" and expression of love (29). In a focus group, mothers from low-income households believed that they were unable to influence their child's weight because it was biologically predetermined (30). In the same study, mothers used descriptive words such as "thick," "solid," or "strong" when describing excess weight (30). parents' beliefs and attitudes toward their childrens' weight status must be considered when implementing nutrition and health interventions for children and families.

Our findings showed that parents' weight was associated with probability of underclassifying their child's weight. Overweight parents may consider their child's overweight or obese status as normal. When overweight and obesity were present in multiple generations within their family, mothers of overweight children perceived their child's weight as normal (29). Studies examining maternal perceptions of young children found inconsistent results regarding parents' BMI and their perception of their child's weight (11,14,18). Our results are consistent with a study of a racially and socioeconomically diverse group of adolescents, indicating higher likelihood of overweight mothers underestimating their adolescent's weight status than underweight mothers (12).

Higher family income was associated with higher probability of parents underclassifying their childrens' weight status. Previous studies that used parent education as an indicator of socioeconomic status found low maternal education to be associated with lower recognition of their child's overweight status (14); however, family income was not used in those studies; thus, direct comparison with our results is not possible. Families with higher income may accept higher weight for their children as a sign of health due to increased food availability. In our study, parents' education was not significantly associated with their perceptions; therefore, this variable was not included in the final model.

A strength of this study is that the findings add to the limited research on parents' attitudes and concerns regarding the weight status of their children among AI populations living on a reservation. All anthropometric data were collected using objective, standardized methods. The results are representative of all children living on this reservation, since more than 95% of children participated. Limitations include the parents' report of sociodemographic data, which could be subject to desirability bias. The cross-sectional design does not allow for causal inferences. Because of the small sample size in some of the weight categories, the results must be interpreted with caution. The findings may not be applicable to other populations of children, especially older, more independent school-age children.

Family participation is essential in obesity prevention and treatment for preschool children (31). Thus, to increase effectiveness of obesity prevention programs, parents must recognize their child's weight status and be concerned about relevant health risks. When parents and other members of the family are overweight, they may be less likely to accept their child's weight issue. Sociocultural factors can influence how parents perceive their child's weight and their behavior toward weight management. If mothers perceive that extra weight for their children adds to their safety and protection, it is possible that with increased family income, higher weight for children is not only acceptable but desirable. Clinicians and interventionists must be aware of these issues and address them when communicating healthy weight management options for preschool children and their families in AI populations.

## Figures and Tables

**Table 1.** Parents' Assessment of Kindergarten child's Weight Status vs child's Measured Weight Status (n = 413) Among American Indians, South Dakota, 2005-2006

Parents' Assessment of Child's Weight Status	Measured Weight Status of Child, n (%)^a^				

	Very Underweight(n = 19)	Underweight(n = 13)	Normal Weight(n = 257)	Overweight (n = 61)	Obese(n = 63)
Very underweight	1 (5.3)	1 (7.7)	1 (0.4)	0	0
Slightly underweight	7 (36.8)	5 (38.5)	21 (8.2)	1 (1.6)	1 (1.6)
Right weight	11 (57.9)	7 (53.8)	231 (89.9)	55 (90.2)	29 (46.0)
Slightly overweight	0	0	4 (1.6)	5 (8.2)	29 (46.0)
Very overweight	0	0	0	0	4 (6.3)

Weight status based on body mass index percentiles from the 2000 CDC Growth Charts (20). Very underweight: <5th percentile; underweight: 5th to <15th percentile; normal weight: 15th to <85th percentile; overweight: 85th to <95th percentile; obese: ≥95th percentile.

**Table 2.** Demographic and Weight Status Characteristics by parents' Assessment of child's Weight Status Among American Indians, South Dakota, 2005-2006^a^

Characteristic	Parent's Classification of Child's Weight, n (%)^b^			*P* Value^c^

	Correctly Classified	Underclassified	Overclassified	
**Child's sex (n = 411)**				
Female	124 (62.0)	59 (29.5)	17 (8.5)	.14
Male	121 (57.3)	79 (37.4)	11 (5.2)	
**Child's age, y (n = 411)**				
4.7 to <6	164 (60.3)	91 (33.5)	17 (6.2)	.80
6 to 7.9	81 (58.3)	47 (33.8)	11 (7.9)	
**BMI-for-age percentile of child (n = 411)**				
<5th	1 (5.6)	0	17 (94.4)	<.001
5th to <15th	5 (38.5)	1 (7.7)	7 (53.8)	
15th to <85th	230 (89.8)	22 (8.6)	4 (1.6)	
85th to <95th	5 (8.2)	56 (91.8)	0	
≥95th	4 (6.3)	59 (93.7)	0	
**Parent's/caregiver's relationship to child (n = 410)**				
Mother/stepmother	165 (59.1)	94 (33.7)	20 (7.2)	.93
Father/stepfather	22 (59.5)	14 (37.8)	1 (2.7)	
Grandmother	36 (58.1)	21 (33.9)	5 (8.1)	
Other	21 (65.6)	9 (28.1)	2 (6.3)	
**Parent's concerns about child's weight (n = 410)**				
Not concerned	185 (64.7)	85 (29.7)	16 (5.6)	.01
A little concerned	35 (46.7)	31 (41.3)	9 (12.0)	
Concerned/very concerned	24 (49.0)	22 (44.9)	3 (6.1)	
**Parent's BMI, kg/m^2^ (n = 411)**				
<25.0 (normal weight)	39 (68.4)	13 (22.8)	5 (8.8)	.08
25.0-29.9 (overweight)	63 (61.8)	29 (28.4)	10 (9.8)	
≥30.0 (obese)	143 (56.7)	96 (38.1)	13 (5.2)	
**Annual household income, $ (n = 401)**				
<15,000	123 (62.4)	62 (31.5)	12 (6.1)	.36
15,000-34,999	77 (59.2)	44 (33.9)	9 (6.9)	
≥35,000	36 (48.6)	31 (41.8)	7 (9.4)	

Abbreviation: BMI, body mass index.

a Sample size may vary because of missing data.

b Correctly classified indicates that parent report of child's weight status is the same as the child's measured weight status; underclassified indicates parent report of child's weight status is lower than measured weight status; overclassified indicates parent report of child's weight status is higher than measured weight status.

c Calculated by using χ^2^ test.

**Table 3.** Associations Between parents' and child's Characteristics and parents' Misclassification of child's Weight Status Among American Indians, South Dakota, 2005-2006

**Characteristic**	Probability of Child Being Underclassified^b^			Probability of Child Being Overclassified^b^		

	n^a^	% (SE)	*P* Value	n^a^	% (SE)	*P* Value
**Child's sex**						
Female	183	31.6 (3.8)	.09	141	10.4 (2.9)	.37
Male	200	40.5 (3.9)		132	7.3 (2.4)	
**Child's age, y**						
4.7 to <6	255	35.5 (3.4)	.74	181	8.0 (2.3)	.40
6 to 7.9	128	37.3 (4.8)		92	10.7 (3.6)	
**Parent's/caregiver's relationship to child**						
Mother/stepmother	259	36.9 (3.4)	.80	185	9.5 (2.5)	.84
Father/stepfather	36	37.1 (8.7)		23	4.3 (4.3)	
Grandmother	57	36.9 (7.0)		41	10.6 (5.1)	
Other	30	27.5 (8.4)		23	7.3 (5.3)	
**Parent's concerns about child's current weight**						
Not concerned	270	31.1 (3.2)^c^	.007	201	6.9 (2.0)	.04
A little concerned	66	48.8 (6.6)		44	19.8 (6.7)	
Concerned/very concerned	46	50.2 (8.2)		27	12.8 (7.2)	
**Parent's BMI, kg/m^2^**						
<25.0 (normal weight)	52	22.4 (6.0)^d^	.02	44	9.6 (4.5)	.66
25.0-29.9 (overweight)	92	31.5 (5.2)		73	11.3 (4.0)	
≥30.0 (obese)	239	41.5 (3.6)		156	7.6 (2.4)	
**Annual household income, %**						
<15,000	185	32.9 (3.8)^e^	.09	135	7.4 (2.4)	.33
15,000-34,999	121	34.6 (4.7)		86	8.6 (3.2)	
≥35,000	67	48.5 (6.6)		43	15.4 (6.0)	

Abbreviations: SE, standard error; BMI, body mass index.

a Sample size may vary because of missing data.

b Correctly classified indicates that parent report of child's weight status is the same as the child's measured weight status; underclassified indicates parent report of child's weight status is lower than measured weight status; overclassified indicates parent report of child's weight status is higher than measured weight status.

c *P* for trend = .02; indicates an increase in the probability of parent underclassifying child's weight status as parent's level of concern increases.

d *P* for trend = .02; indicates an increase in the probability of parent underclassifying child's weight status as parent's own BMI increases.

e *P* for trend = .04; indicates an increase in the probability of parent underclassifying child's weight status as parent's income level increases.
